# A Pseudomonas aeruginosa Antimicrobial Affects the Biogeography but Not Fitness of Staphylococcus aureus during Coculture

**DOI:** 10.1128/mBio.00047-21

**Published:** 2021-03-30

**Authors:** Juan P. Barraza, Marvin Whiteley

**Affiliations:** a School of Biological Sciences, Georgia Institute of Technology, Atlanta, Georgia, USA; b Center for Microbial Dynamics and Infection, Georgia Institute of Technology, Atlanta, Georgia, USA; c Emory–Children’s Cystic Fibrosis Center, Atlanta, Georgia, USA; University of Iowa; Harvard Medical School

**Keywords:** *Pseudomonas aeruginosa*, *Staphylococcus aureus*, model, HQNO, spatial structure, cystic fibrosis, SCFM2, biogeography, coculture, model system

## Abstract

Pseudomonas aeruginosa and Staphylococcus aureus are two of the most common coinfecting bacteria in human infections, including the cystic fibrosis (CF) lung. There is emerging evidence that coinfection with these microbes enhances disease severity and antimicrobial tolerance through direct interactions. However, one of the challenges to studying microbial interactions relevant to human infection is the lack of experimental models with the versatility to investigate complex interaction dynamics while maintaining biological relevance. Here, we developed a model based on an *in vitro* medium that mimics human CF lung secretions (synthetic CF sputum medium [SCFM2]) and allows time-resolved assessment of fitness and community spatial structure at the micrometer scale. Our results reveal that P. aeruginosa and S. aureus coexist as spatially structured communities in SCFM2 under static growth conditions, with S. aureus enriched at a distance of 3.5 μm from P. aeruginosa. Multispecies aggregates were rare, and aggregate (biofilm) sizes resembled those in human CF sputum. Elimination of P. aeruginosa’s ability to produce the antistaphylococcal small molecule HQNO (2-heptyl-4-hydroxyquinoline *N*-oxide) had no effect on bacterial fitness but altered the spatial structure of the community by increasing the distance of S. aureus from P. aeruginosa to 7.6 μm. Lastly, we show that coculture with P. aeruginosa sensitizes S. aureus to killing by the antibiotic tobramycin compared to monoculture growth despite HQNO enhancing tolerance during coculture. Our findings reveal that SCFM2 is a powerful model for studying P. aeruginosa and S. aureus and that HQNO alters S. aureus biogeography and antibiotic susceptibility without affecting fitness.

## Abstract (precis)

Many human infections result from the action of multispecies bacterial communities. Within these communities, bacteria have been proposed to directly interact via physical and chemical means, resulting in increased disease and antimicrobial tolerance.

## INTRODUCTION

Polymicrobial infections often cause more damage and are more recalcitrant to clearance than those caused by a single microbe (1–5). Two bacteria commonly found together in human polymicrobial infections are Pseudomonas aeruginosa and Staphylococcus aureus, which cause chronic infections at a number of body sites in individuals with a variety of comorbidities, including cystic fibrosis (CF) (6, 7). P. aeruginosa and S. aureus are the two most common bacteria infecting the CF lung, and their coinfection is associated with increased morbidity and mortality (8–11). Experiments in animal models coinoculated with P. aeruginosa and S. aureus indicate that coinfection increases disease severity and antimicrobial resistance (12–16).

While animal models have provided insights into P. aeruginosa-S. aureus coinfections, in many cases the molecular mechanisms controlling enhanced pathogenesis and antimicrobial resistance are not known. One of the challenges to defining coinfection mechanisms is that animal models of infections are constrained in model design, with regard to both the numbers of bacteria required for establishing an infection and the duration of the infection. In addition, time-resolved, simultaneous assessment of bacterial fitness, spatial structure, and function is often not feasible in animal models. This has necessitated the development of versatile, *in vitro* models to discover and molecularly characterize P. aeruginosa-S. aureus coculture interaction mechanisms, which can subsequently be studied in animal models. However, developing *in vitro* experimental models has been challenging, as P. aeruginosa is highly lytic for S. aureus under most *in vitro* coculture conditions (15–23). As a consequence, cocultures are generally stable only when P. aeruginosa is at low cell density (22). Given these challenges, work to mechanistically characterize interactions between P. aeruginosa and S. aureus has often been performed by exposing one bacterium to the cell-free supernatant of the other (24–27). These studies have shown that P. aeruginosa antistaphylococcal activity is driven by exoproducts, including proteases and secondary metabolites such as hydrogen cyanide, phenazines, and quinoline *N*-oxides (18, 23, 28–30).

One of the most widely recognized exoproducts of P. aeruginosa with potent antistaphylococcal activity is 2-heptyl-4-hydroxyquinoline *N*-oxide (HQNO). HQNO has been found in CF lung exudates and kills S. aureus by inhibiting cellular respiration and reducing cellular ATP (23, 31, 32). In addition to its potent antistaphylococcal activity, sublytic levels of HQNO can alter the physiology of S. aureus by shifting its metabolism from respiration to fermentation (33), increasing biofilm formation (27), inducing a small-colony variant phenotype (34), and increasing its susceptibility to membrane-targeting antimicrobials, (25) while decreasing its susceptibility to aminoglycosides (24).

While supernatant addition experiments have identified HQNO and other potential interaction mechanisms driven by secreted products, they do not allow study of cell-cell interactions or the spatial organization of the microbial community, both of which impact polymicrobial infection outcomes (35–39). Thus, there is a need for experimental models that allow for stable coculture of P. aeruginosa and S. aureus in the laboratory. Several studies have developed such systems by altering the bacterial genotype or growth conditions. For example, *in vitro* coexistence has been obtained by using a mucoid strain of P. aeruginosa that has less lytic activity against S. aureus (40), by exchanging medium and removing planktonic cells to extend coexistence (33), or by altering the frequency of P. aeruginosa/S. aureus or the growth environment (20, 41). However, laboratory coculture models can be further improved with the explicit goal of mimicking both the chemical and physical environment of the human infection site (2, 42).

In this study, we combined ecological and molecular techniques to understand interactions between P. aeruginosa and S. aureus in an *in vitro* infection model (synthetic CF sputum medium [SCFM2]) that has been shown to mimic the chemical and physical environment of expectorated CF sputum (17, 21, 43–45). We demonstrated that S. aureus and P. aeruginosa robustly coexist in SCFM2 under static conditions but not in mixed coculture. Then, using high-resolution confocal microscopy and a computational framework that quantifies spatial structure at the micrometer scale, we found that HQNO can alter spatial patterning between the two species without altering fitness. Further, we showed that HQNO increases tolerance of S. aureus to the aminoglycoside tobramycin in coculture with P. aeruginosa.

## RESULTS AND DISCUSSION

### P. aeruginosa and S. aureus coexist in static but not well-mixed SCFM2.

The goal of this study was to develop a biologically relevant *in vitro* experimental system that allows the coexistence of P. aeruginosa and S. aureus and provides the versatility to study their interactions with micrometer-scale spatial resolution. The system we chose was coculture in SCFM2, a defined medium designed by quantifying the chemical composition of sputum expectorated by individuals with CF (45). The gene expression signature of P. aeruginosa grown in SCFM2 is more similar to that in CF sputum directly harvested from humans than other CF preclinical models, including a mouse acute lung model (43). P. aeruginosa also requires similar genes to grow in SCFM2 and *ex vivo* in expectorated human CF sputum (45). Importantly for this study, SCFM2 contains relevant levels of DNA and mucin, which promotes the natural formation of P. aeruginosa aggregates with sizes similar to those observed in the CF lung (17, 44). SCFM2 has also been shown to be a valuable model for studying S. aureus CF infection, including understanding how host immune components affect S. aureus physiology and gene expression (9, 42). Of note, growth of both P. aeruginosa and S. aureus in SCFM2 has been performed previously without mixing under static growth conditions (17, 42, 44, 45).

To determine whether these bacteria can stably coexist in SCFM2, laboratory strains of P. aeruginosa (PA14) and S. aureus (LAC) were coinoculated into SCFM2 at a 1:1 frequency, cultures were incubated statically or mixed with a magnetic stir bar, and bacterial numbers were quantified at 4-h intervals using agar plate counts on selective media. These strains are well-characterized laboratory strains, are highly virulent and antagonistic, have been used to study P. aeruginosa-S. aureus interactions (12, 40, 46, 47), and are representative of other pathogenic strains of the same species, thus incorporating clinical relevance into the study. In addition, these strains display gene expression patterns and aggregate sizes in SCFM2 that are similar to those in human expectorated CF sputum (17, 42–44, 48). As these are not highly adapted CF strains, they serve as a model to study potential interactions in early CF disease. There is no doubt that using adapted CF strains would be more relevant. However, our recent studies show that gene expression of CF-adapted strains in SCFM2 is only slightly more representative of that in later CF disease than that of lab strains (43). Thus, our studies will likely have some relevance for understanding later-stage disease.

**FIG 1:**
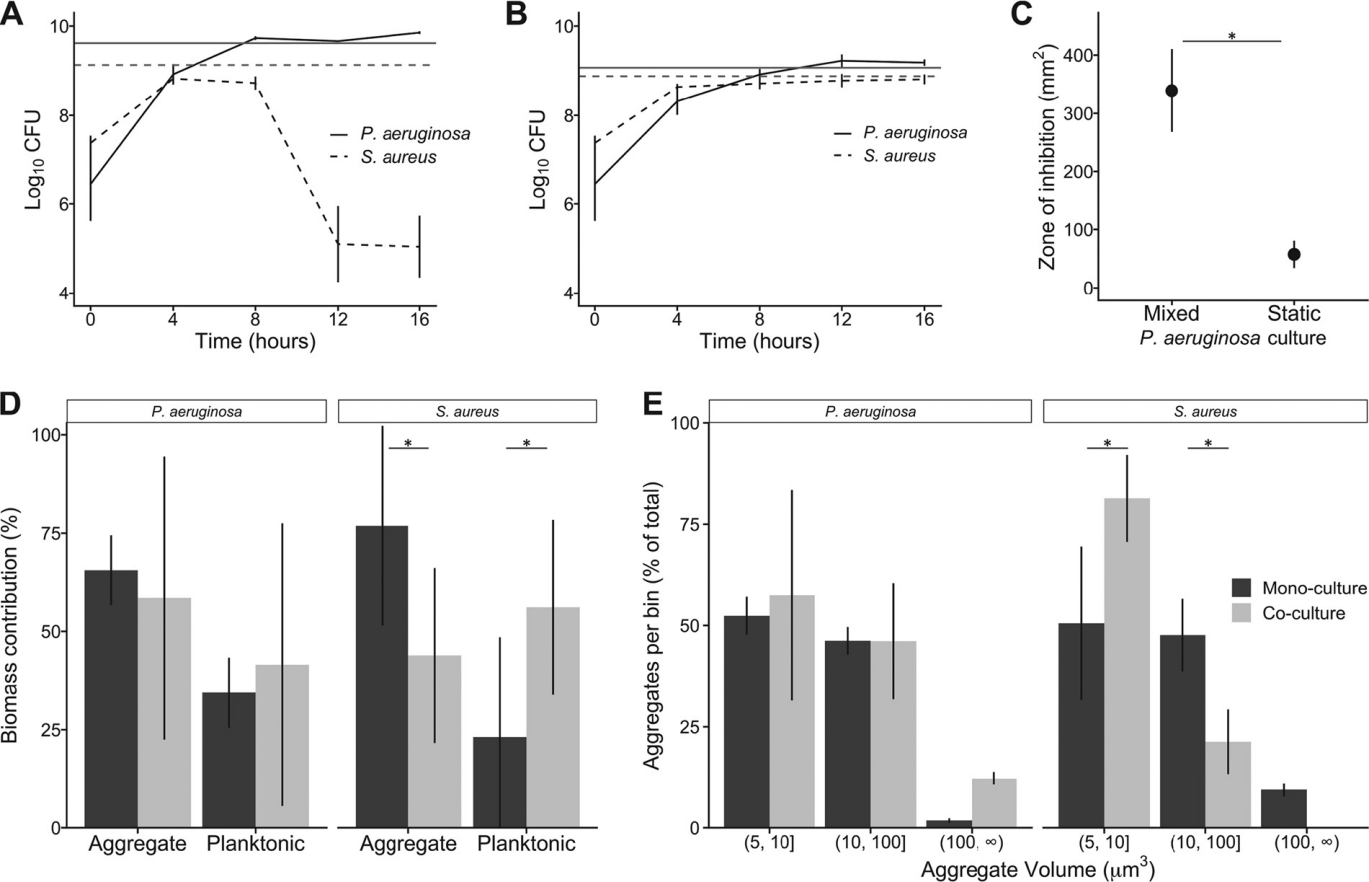
S. aureus and P. aeruginosa stably coexist in static SCFM2. Growth of P. aeruginosa PA14 and S. aureus LAC under (A) well-mixed and (B) static conditions in SCFM2 (*n* = 3). Black lines represent CFU in coculture, and the horizontal dark gray lines indicate growth yield after 16 h in monoculture. (C) Normalized zone of inhibition produced by P. aeruginosa supernatants spotted on filter discs on lawns of S. aureus. P. aeruginosa produced a larger zone of inhibition when grown well-mixed in SCFM2 than when grown statically in SCFM2 (*n* = 5, paired Student's *t* test, *P* = 1 × 10^−3^). (D) Aggregate and planktonic biomass of P. aeruginosa and S. aureus in SCFM2 in mono- and coculture. S. aureus biomass primarily exists as aggregates in monoculture and as planktonic cells in coculture. Black bars represent monoculture, and gray bars represent coculture (*n* = 3, paired Student's *t* test, *P* = 0.02 for both comparisons). (E) Number of aggregates of P. aeruginosa and S. aureus within different aggregate size ranges in mono- and coculture. We quantified the number of aggregates in three size ranges: 5 μm^3^ to 10 μm^3^, 10 μm^3^ to 100 μm^3^, and larger than 100 μm^3^ and reported the percentage of total aggregates in each size range. A lower percentage of aggregates were observed in the 5- to 10-μm^3^ range during monoculture than during coculture (50% versus 80%) for S. aureus (*n* = 3, paired Student's *t* test, *P* = 0.05), and a correspondingly higher percentage of aggregates in the 10- to 100-μm^3^ range were observed in coculture than in monoculture (20% versus 47%) (paired Student's *t* test, *P* = 0.03). Black bars represent monoculture, and gray bars represent coculture. Error bars show standard deviations. (*, *P* < 0.05, paired Student's *t* test.).

Our results reveal that, as previously observed, P. aeruginosa is highly lytic for S. aureus in well-mixed cocultures, reducing S. aureus numbers by ∼10,000-fold between hours 8 and 12 (Fig. 1A). However, this decrease in S. aureus numbers was not observed when cocultures were incubated statically (Fig. 1B). Moreover, both P. aeruginosa and S. aureus grew to a density in static coculture similar to that in static monoculture (Fig. 1). These results indicate that P. aeruginosa and S. aureus coexist in SCFM2 when grown under static conditions with no loss of fitness compared to monoculture static growth.

### Antistaphylococcal activity in P. aeruginosa is higher under well-mixed culture conditions.

P. aeruginosa produces several secreted antistaphylococcal molecules, and many of these molecules are produced at higher levels in the presence of oxygen (49, 50). Thus, we hypothesized that a primary mechanism promoting stable P. aeruginosa-S. aureus coculture under static conditions was decreased production of antistaphylococcal molecules by P. aeruginosa due to decreased mixing. To test this hypothesis, cell-free supernatants from P. aeruginosa grown in SCFM2 under mixed and static conditions were collected and assessed for the ability to inhibit S. aureus growth using a disc diffusion assay (Fig. 1C). For this assay, P. aeruginosa supernatant was added to a filter disc on an agar plate containing S. aureus, and the zone of inhibition was measured. P. aeruginosa grown as a well-mixed culture in SCFM2 produced a zone of inhibition with a diameter more than ∼6 times larger than growth in static culture (Fig. 1C). These results reveal that P. aeruginosa supernatants from well-mixed cultures possess higher antistaphylococcal activity than those from static cultures.

Our findings that ecological factors (well-mixed and static growth) affect P. aeruginosa antistaphylococcal activity and the outcome of P. aeruginosa-S. aureus coculture dynamics are fundamentally in agreement with recent work from Niggli and Kummerli (20), which showed that P. aeruginosa and S. aureus coexist in a laboratory medium that promotes aggregation by embedding in low levels of agar. However, that study did not observe an increase in P. aeruginosa relative fitness compared to S. aureus during growth in mixed conditions compared to agar conditions, which differs from our observations (Fig. 1A and B). The reason for this is not clear, but it is likely explained by low aeration of the mixed culture condition used by Niggli and Kummerli (20). That study performed mixed coculture in laboratory medium in wells of 24-well plates, using a volume of 1.5 ml in a well with a maximum volume of 3.4 ml and shaking at 170 rpm (20). In our experience, P. aeruginosa requires high shaking rates (250 rpm) and low culture volume/culture vessel volume (1/10 to 1/50) for sufficient aeration, and these culture conditions lead to high antistaphylococcal activity (15, 19, 21, 22). Here, we mixed SCFM2 using a stir bar at 250 rpm to ensure high levels of aeration. Regardless, both studies agree that P. aeruginosa and S. aureus coexist in culture conditions that promote aggregation, which restricts movement and promotes the development of spatial structure.

### Aggregate sizes and distributions in P. aeruginosa-S. aureus mono- and cocultures.

**FIG 2:**
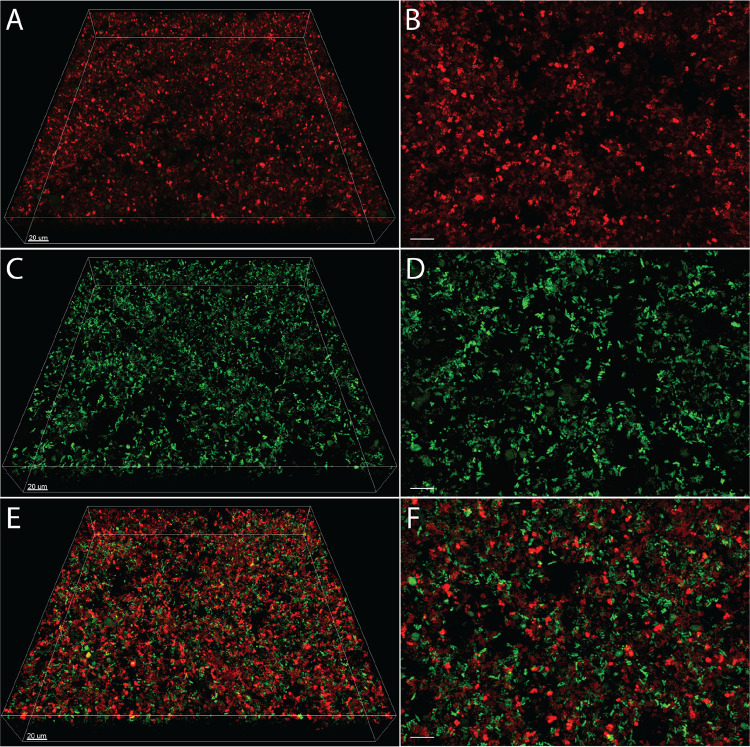
Images of P. aeruginosa and S. aureus in mono- and coculture in SCFM2. Representative confocal images of DsRed-expressing S. aureus LAC (red) in (A and B) monoculture, GFP-expressing P. aeruginosa PA14 (green) in monoculture (C and D), and S. aureus and P. aeruginosa in coculture (E and F). Images on the left (A, C, and E) show the entire imaging field of 270 μm by 270 μm by 40 μm. Images on the right (B, D, and F) show a close-up view of images on the left. Bars, 10 μm unless otherwise noted.

The micrometer-scale spatial structure of infecting polymicrobial communities has been shown to affect infection severity in a mouse abscess model (39); thus, one of the goals of this work was to develop a biologically relevant experimental system that allows the spatial structure of P. aeruginosa and S. aureus to be assessed temporally and at the micrometer scale. Based on the diversity of interactions that have been described, we hypothesized that there would be significant differences in spatial structure of P. aeruginosa-S. aureus cocultures compared to monoculture, despite the fact that cell numbers are equivalent (Fig. 1B). To test this hypothesis, we inoculated P. aeruginosa expressing the green fluorescent protein (GFP) and S. aureus expressing the red fluorescent protein DsRed in mono- and coculture (1:1 frequency) into SCFM2, incubated them statically, and imaged them using confocal laser scanning microscopy (CLSM) (Fig. 2). We chose to end our experiments at 5 h for several reasons: (i) transcriptomic analysis of P. aeruginosa at the 5- to 6-h time point in SCFM2 has been shown to most accurately mimic the gene expression of P. aeruginosa in human expectorated sputum (43, 48); (ii) transcriptomic analysis of S. aureus in SCFM2 at this time point is also similar to that in human expectorated sputum (42); (iii) P. aeruginosa naturally forms aggregates in SCFM2 with sizes similar to those in expectorated CF sputum at this time point (17); (iv) S. aureus and P. aeruginosa numbers at this time point are within the range often observed in human CF sputum; and (v) S. aureus has just reached maximum growth yields at this time point, and DsRed fluorescence fades rapidly as the cells progress deeper into stationary phase. This may be caused by a transcriptional regulation of the *sarA* promoter, which drives DsRed (51).

To quantify the spatial structure of each culture, we utilized a custom computational pipeline (publicly available at https://jupabago.github.io/PaSaProject/) that was recently used to quantify the spatial structure of bacterial communities on human teeth (37). The first step in this pipeline is to discriminate between bacterial cells growing planktonically and those growing as aggregates (biofilms). To accomplish this, we first identified all bacterial objects from CLSM images and classified them by volume, using previously established guidelines defining planktonic cells as objects with a size of <5 μm^3^ and aggregates as those with a size of ≥5 μm^3^ (17). This analysis revealed that S. aureus and P. aeruginosa are present as both planktonic cells and aggregates in mono- and coculture in SCFM2 (Fig. 1D). However, the percentage of S. aureus biomass in aggregates in monoculture (∼75%) was twice as high as in coculture, and correspondingly, planktonic cells contributed less to the total biomass in mono- than coculture (Fig. 1D). In contrast, aggregates of P. aeruginosa in mono- and coculture contributed equally to total biomass, although the coculture values displayed higher variance (Fig. 1D).

Next, we focused only on the portion of the biomass in aggregates to determine whether coculture impacted aggregate size. We defined bins of increasing aggregate size and quantified the number of P. aeruginosa and S. aureus aggregates within each bin (Fig. 1E). The bin sizes were chosen to include the most common observed aggregate size range in human expectorated sputum (10 to 100 μm^3^) as well as a smaller and a larger bin (17, 52–54). P. aeruginosa aggregate size was not affected by the presence of S. aureus, with over 95% of aggregates being ≤100 μm^3^ in both mono- and coculture. However, S. aureus had a higher percentage of aggregates that were ≤10 μm^3^ in coculture compared to monoculture (80% versus 50%, respectively) and a correspondingly lower percentage that were between 10 and 100 μm^3^ (20% versus 47%).

Collectively, these results reveal that while P. aeruginosa and S. aureus both exist as aggregates and planktonic cells in mono- and coculture, the S. aureus population shifts toward planktonic cells and small aggregates during coculture. The biological relevance of the shift of S. aureus to a more planktonic mode during coculture is not known. However, as there is no decrease in fitness in coculture compared to monoculture under static growth conditions (Fig. 1B), it is clear that it is not necessary for S. aureus to grow as large aggregate biofilms to be fit in the presence of P. aeruginosa. The finding that P. aeruginosa exists as both aggregates (65% biomass in monoculture) (Fig. 1D) and planktonic cells (35% biomass in monoculture) (Fig. 1D) in SCFM2 further supports the biological relevance of this model, as recent studies revealed that P. aeruginosa exists in expectorated CF sputum as both aggregates (∼75% of biomass) and planktonic cells (∼25% of biomass) (17, 52, 54).

### Impact of HQNO on P. aeruginosa-S. aureus community structure.

As we have now developed a biologically relevant coculture model for studying P. aeruginosa-S. aureus interactions, we next sought to examine the impact of P. aeruginosa-produced HQNO on this community. We chose HQNO as not only does it have antimicrobial activity against S. aureus, but also, subinhibitory levels impact the physiology of S. aureus, including susceptibility to antimicrobials (24, 25, 27, 33, 34). In addition, the gene encoding the enzyme required for the final step in HQNO biosynthesis (*pqsL*) is expressed similarly in static SCFM2 at the 5-h time point and in human expectorated sputum (43), providing evidence of the biological relevance of SCFM2 for studying HQNO at this time point.

**FIG 3:**
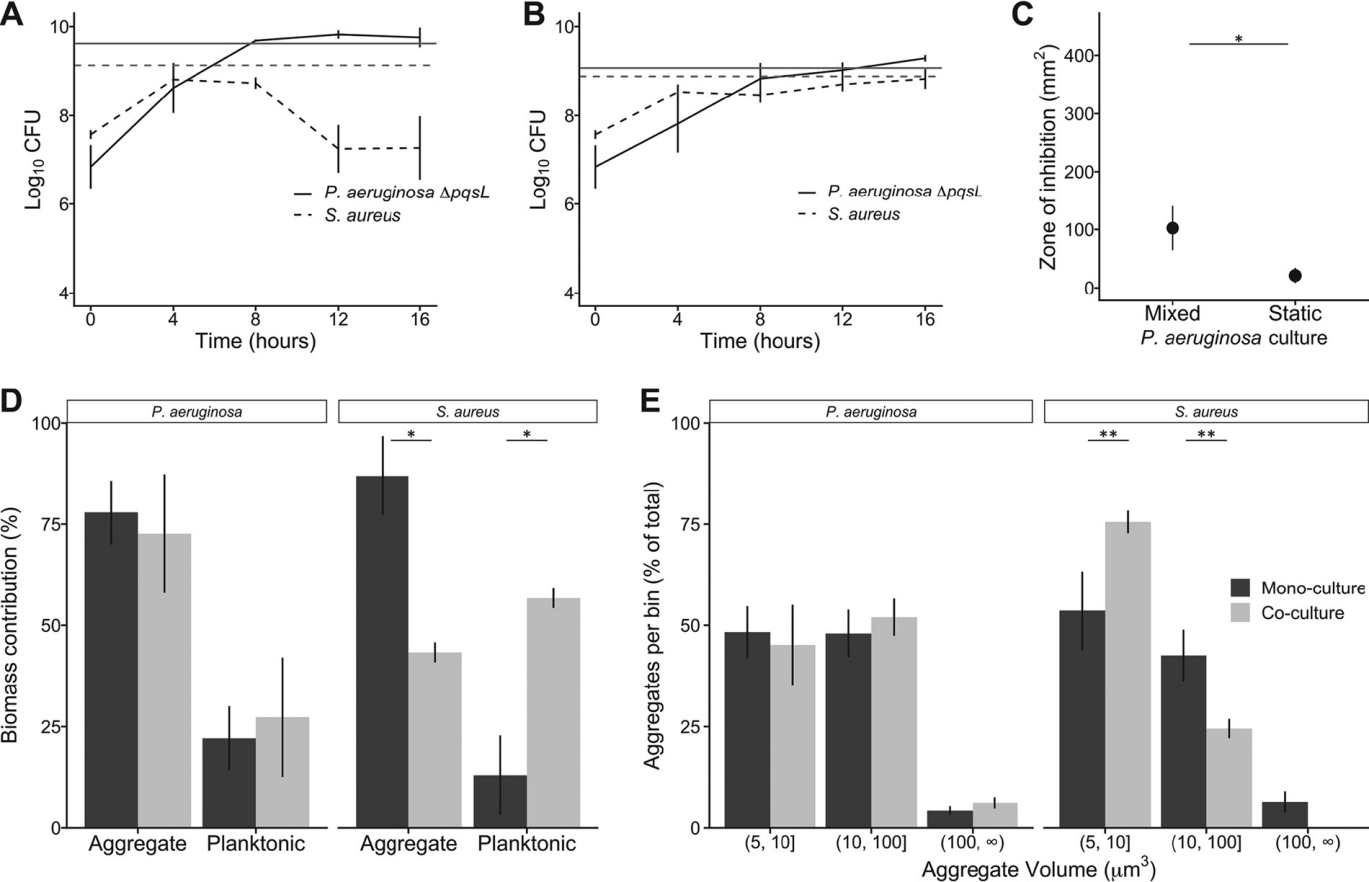
HQNO impacts S. aureus fitness in well-mixed but not static SCFM2 cocultures. Growth of P. aeruginosa Δ*pqsL* and S. aureus under (A) well-mixed and (B) static conditions in SCFM2 (*n* = 3). Black lines represent CFU over time in coculture, and the horizontal dark gray line indicates growth yield after 16 h in monoculture. (C) Zone of inhibition produced by P. aeruginosa Δ*pqsL* supernatants spotted on filter discs on lawns of S. aureus. P. aeruginosa produced a larger zone of inhibition when grown well-mixed in SCFM2 than in static SCFM2 (*n* = 5, paired Student's *t* test, *P* = 0.012) but not as large as wild-type P. aeruginosa (*P* = 0.023; Fig. 1C). (D) Aggregate and planktonic biomass of P. aeruginosa Δ*pqsL* and S. aureus in SCFM2 mono- and coculture. Similar to coculture with wild-type P. aeruginosa, S. aureus biomass primarily exists as aggregates in monoculture and as planktonic cells in coculture (paired Student's *t* test, *P* = 0.02). P. aeruginosa Δ*pqsL* monoculture biomass was also found to be more present as aggregates than as planktonic cells (paired Student's *t* test, *P* = 0.02). (E) Number of aggregates of P. aeruginosa Δ*pqsL* and S. aureus within different aggregate size ranges in mono- and coculture. We quantified the number of aggregates in three size ranges (5 to 10 μm^3^, 10 to 100 μm^3^, and larger than 100 μm^3^) and reported the percentage of total aggregates in each size range. Fewer aggregates were observed in the 5- to 10-μm^3^ range during monoculture than in coculture for S. aureus (*n* = 3, paired Student's *t* test, *P* = 0.08), and a corresponding higher percentage of aggregates in the 10- to 100-μm^3^ range were observed in the monoculture than in coculture (*n* = 3, paired Student's *t* test, *P* = 0.08). Error bars show standard deviations (*, *P* < 0.05; **, *P* < 0.1 [paired Student's *t* test]).

To examine the role of HQNO in community structure, we first created a strain of P. aeruginosa that does not produce HQNO by deleting *pqsL* (P. aeruginosa Δ*pqsL*) and showed that complementation of this strain with *pqsL* in *trans* restored S. aureus lytic ability (see Fig. S1 in the supplemental material). Next, we cocultured S. aureus under mixed and static conditions with P. aeruginosa Δ*pqsL*. Under well-mixed conditions, P. aeruginosa Δ*pqsL* lysed S. aureus, but to a lesser degree than wild-type P. aeruginosa, reducing S. aureus numbers by ∼100-fold between 8 and 12 h (Fig. 3A). Under static growth conditions, P. aeruginosa Δ*pqsL* and S. aureus coexisted and reached similar growth yields in both mono- and coculture (Fig. 3B). As expected from the decrease in S. aureus levels at late stages of growth, supernatants from P. aeruginosa Δ*pqsL* grown as well-mixed cultures exhibited antistaphylococcal activity against S. aureus, as observed using the disc diffusion assay (Fig. 3C). This activity was less than that observed for well-mixed wild-type P. aeruginosa (*P* = 0.023, Student's *t* test) and similar to that observed for supernatants from static wild-type P. aeruginosa. However, supernatants from P. aeruginosa Δ*pqsL* grown statically had little antimicrobial activity (Fig. 3C). These data reveal that the antistaphylococcal activity of HQNO has biological importance in well-mixed, but not static, coculture conditions and that HQNO is not the only lytic factor in well-mixed SCFM2 cocultures.

10.1128/mBio.00047-21.1 FIG S1 Expression of *pqsL* in *trans* restores S. aureus lytic activity in P. aeruginosa PA14 Δ*pqsL*. (A) Image of BHI agar plate showing zones of inhibition formed by P. aeruginosa PA14, P. aeruginosa PA14 Δ*pqsL*, P. aeruginosa PA14 Δ*pqsL* carrying the complementation plasmid pBBR1-pqsL, and P. aeruginosa PA14 Δ*pqsL* carrying the control plasmid pBBR1MCS-5 (labeled pBBR1, empty vector) on an S. aureus LAC lawn. (B) Zone of inhibition (in square millimeters) produced by each strain. Error bars show standard deviations (*n* = 4). *, *P* < 10^−3^ using a one-way analysis of variance (ANOVA) with Tukey’s multiple-comparison test. Download FIG S1, TIF file, 2.2 MB. Copyright © 2021 Barraza and Whiteley. 2021 Barraza and Whiteley. https://creativecommons.org/licenses/by/4.0/ This content is distributed under the terms of the Creative Commons Attribution 4.0 International license.

### Aggregate sizes and distributions in P. aeruginosa Δ*pqsL* and S. aureus mono- and cocultures.

**FIG 4:**
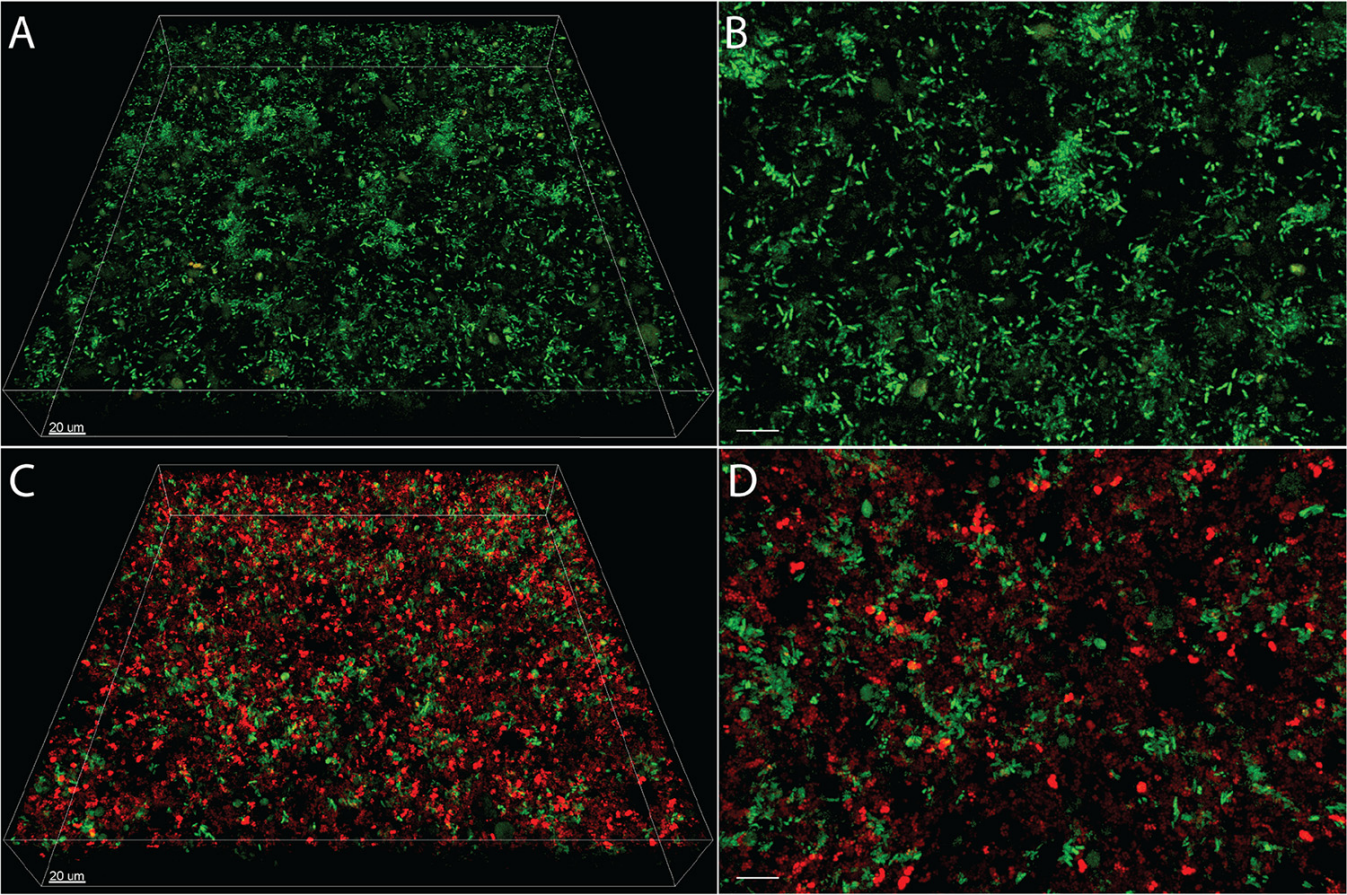
Images of P. aeruginosa Δ*pqsL* in mono- and coculture with S. aureus in SCFM2. Representative confocal images of GFP-expressing P. aeruginosa Δ*pqsL* PA14 (green) in (A and B) monoculture and (C and D) coculture with DsRed-expressing S. aureus LAC (red). Images on the left (A and C) show the entire imaging field of 270 μm by 270 μm by 40 μm. Images on the right (B and D) show a close-up view of images on the left. Bars, 10 μm unless otherwise noted.

While HQNO had no effect on S. aureus fitness during static coculture, we next assessed whether this molecule impacted P. aeruginosa Δ*pqsL* and S. aureus aggregate number and size using confocal microscopy (Fig. 4), as described above for wild-type P. aeruginosa-S. aureus cocultures (Fig. 1D and E). This analysis revealed that cocultures containing P. aeruginosa Δ*pqsL* (Fig. 3D) were overall similar to those with wild-type P. aeruginosa (Fig. 1D) in regard to aggregate biomass, with P. aeruginosa Δ*pqsL* primarily being found as aggregates in both monoculture (77%) and coculture (72%) and S. aureus existing primarily as aggregates in monoculture and as planktonic cells in coculture (Fig. 3D). Similar to wild-type P. aeruginosa, P. aeruginosa Δ*pqsL* aggregate size was not affected by the presence of S. aureus, with over 95% of aggregates being ≤100 μm^3^ in both mono- and coculture (Fig. 3E). In addition, S. aureus had a higher percentage of aggregates that were ≤10 μm^3^ in coculture than in monoculture (75% versus 55%, respectively) and a correspondingly lower percentage that were between 10 and 100 μm^3^ (25% versus 40%, respectively). These results reveal that although HQNO is an important contributor to S. aureus lysis during well-mixed coculture, it plays no role in P. aeruginosa and S. aureus aggregate biomass and size during static coculture.

### HQNO impacts spatial organization of P. aeruginosa and S. aureus cocultures.

While HQNO had no detectable effect on biomass or aggregate size during coculture, we hypothesized that due to its antimicrobial activity, this molecule would impact the spatial organization of the community by increasing the distance between P. aeruginosa and S. aureus. To test this hypothesis, we quantified spatial organization of P. aeruginosa-S. aureus cocultures using two metrics: coaggregation and enrichment distance.

**FIG 5:**
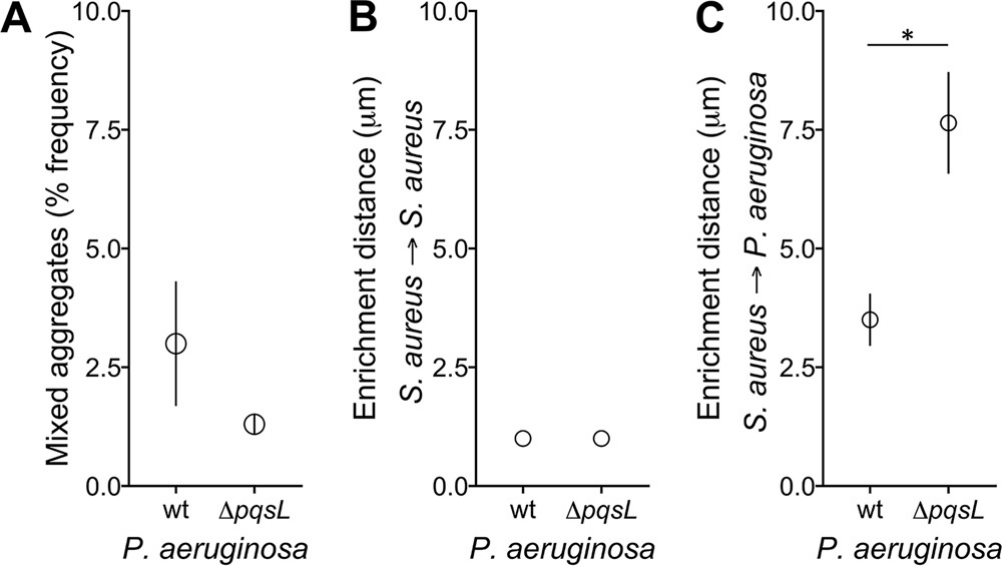
HQNO impacts the spatial organization of P. aeruginosa and S. aureus communities. (A) Percent mixed-species aggregates of S. aureus with P. aeruginosa wild-type (wt) and Δ*pqsL* during static growth in SCFM2. (B) Enrichment distance calculated using S. aureus as both the focal species and target species, indicating that S. aureus is most often found tightly associated with other S. aureus cells. (C) Enrichment distance calculated using S. aureus as the focal species and P. aeruginosa as the target species. S. aureus was localized closer to wild-type P. aeruginosa than P. aeruginosa Δ*pqsL* (paired Student's *t* test, *P* = 0.01). Error bars show standard deviations.

Coaggregation is a common occurrence in many microbial systems and can be quantified by counting the prevalence of aggregates that contain multiple species (55). To test for coaggregation in the P. aeruginosa-S. aureus SCFM2 static cocultures, we quantified the proportion of aggregates that contain both P. aeruginosa *and* S. aureus. Our results reveal that coaggregation does not constitute a significant portion of the total biomass in cocultures containing either wild-type P. aeruginosa or P. aeruginosa Δ*pqsL*, with 1 to 3% of the total aggregates containing both P. aeruginosa and S. aureus (Fig. 5A). These data indicate that wild-type P. aeruginosa and S. aureus do not produce substantial numbers of mixed-species aggregates in SCFM2, and the elimination of HQNO does not impact the prevalence of mixed aggregates. These data are also consistent with previous data examining coaggregation of P. aeruginosa PA14 strains that express different fluorescent proteins, which revealed that P. aeruginosa aggregates primarily arise from single cells (44).

We next asked whether P. aeruginosa and S. aureus were randomly distributed in SCFM2 or if there was spatial patterning. To answer this question, we characterized spatial patterning of P. aeruginosa-S. aureus aggregates by calculating enrichment distance. To determine this metric, we first calculated proportional occupancy, which quantifies the composition of the immediate surroundings of a focal community member in relation to other community members at various distance intervals in three dimensions at the micrometer scale (37). Then, enrichment distance was defined as the distance from the focal species at which the proportional occupancy of the target species is the highest. Thus, enrichment distance indicates where biomass of the target species is overrepresented in relation to the focal species. We calculated enrichment distance for cocultures using S. aureus as the focal species and S. aureus or P. aeruginosa as the target species, for both wild-type P. aeruginosa and P. aeruginosa Δ*pqsL*. In each case, we chose five thousand random DsRed voxels corresponding to S. aureus for each replicate and calculated the prevalence of target species voxels within defined distance intervals (30 intervals, each 1 μm). Our results reveal that the enrichment distance of S. aureus to S. aureus was 0 to 1 μm when cocultured with either the wild-type or Δ*pqsL* strain (Fig. 5B), which is the smallest distance interval tested. These results indicate that on average, an S. aureus cell is most often located within 1 μm of a second S. aureus cell. These results make intuitive sense and serve as a control for our metric, as a proportion of S. aureus in these communities exists in aggregates, which are by definition tightly associated groups of cells. The enrichment distance of S. aureus to wild-type P. aeruginosa was 3.5 μm, indicating that during static coculture in SCFM2, these two bacteria exist in close proximity to one another (Fig. 5C). Intriguingly, S. aureus was on average found at a further distance from P. aeruginosa Δ*pqsL* (7.6 μm) than from wild-type P. aeruginosa. Thus, although HQNO does not impact the fitness S. aureus and P. aeruginosa (Fig. 1 and 3), it does affect the spatial structure of the community.

These results are surprising, as we expected that elimination of a potent antimicrobial would allow closer association between P. aeruginosa and S. aureus. While the mechanism(s) underlying this phenotype is not known, one simple model is competition for molecular oxygen as an electron acceptor. P. aeruginosa predominantly respires to generate energy, and while there are low levels of nitrate in SCFM2 that can be used (350 μM), significantly higher levels (50 mM) are needed to support high-yield growth of P. aeruginosa in SCFM (56). Thus, O_2_ is likely the predominant electron acceptor used during growth in SCFM2. HQNO has been shown to shift S. aureus metabolism from respiration to fermentation (33), which may allow close colocalization by preventing competition for O_2_. Elimination of HQNO would likely cause competition for O_2_, which would lead to P. aeruginosa growing in locations where O_2_ levels are higher, further from S. aureus. Testing this model will require, among other approaches, a technological advancement in micrometer-scale oxygen measurement, which we are currently pursuing using electrochemical approaches.

### HQNO enhances tobramycin resistance in S. aureus during coculture, although monocultures of S. aureus are still more resistant.

**FIG 6:**
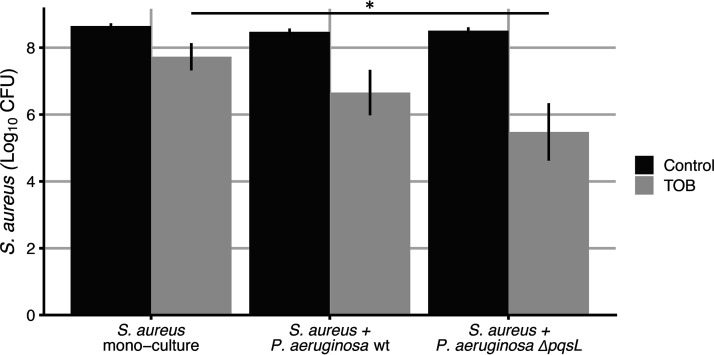
HQNO enhances S. aureus survival to tobramycin during coculture. S. aureus was grown in SCFM2 under three conditions: monoculture, coculture with P. aeruginosa, and coculture with P. aeruginosa Δ*pqsL*. Cultures were then treated with tobramycin (256 μg/ml) or water (control), and the number of S. aureus CFU was determined. (*n* = 12; *, *P <* 0.05 by the Kruskal-Wallis test, followed by a *post hoc* paired Wilcoxon test). Error bars indicate standard deviations.

Previous studies have shown that the presence of HQNO in P. aeruginosa supernatants enhances aminoglycoside resistance in S. aureus (26). Thus, we used our system to assess whether these findings are also observed in cocultures. We incubated S. aureus statically in mono- or coculture with P. aeruginosa in SCFM2 for 3 h and then treated the culture with a level of tobramycin (256 μg/ml) that results in 90% killing of monoculture S. aureus grown statically in SCFM2 (Fig. 6). Coculture with either wild-type P. aeruginosa or the Δ*pqsL* strain increased S. aureus susceptibility to tobramycin compared to monoculture (Fig. 6). Further, this increased susceptibility was greatest in coculture with the Δ*pqsL* mutant, which showed a 10-fold decrease in S. aureus numbers relative to coculture with wild-type P. aeruginosa. These results reveal that similar to previous experiments with P. aeruginosa supernatants, HQNO enhances tobramycin resistance of S. aureus during coculture with P. aeruginosa. However, S. aureus monocultures are significantly more resistant to tobramycin killing than cocultures with P. aeruginosa, suggesting that ultimately, P. aeruginosa sensitizes S. aureus to tobramycin killing even in the presence of HQNO. These data are also consistent with recent high-throughput S. aureus mutant experiments, which show that P. aeruginosa imparts significant stress to S. aureus in coinfected murine wounds (13).

### Conclusions.

Our studies reveal that static growth in SCFM2 allows long-term coculture of S. aureus with a strain of P. aeruginosa that has high antistaphylococcal activity under well-mixed conditions. Based on this study and our previous findings that SCFM2 is a biologically relevant model for studying CF lung infections (17, 42–45), we propose that this coculture model provides a means to study interactions between S. aureus, P. aeruginosa, and potentially other bacteria infecting the CF lung. Our results also reveal that elimination of HQNO has no effect on the fitness of S. aureus or P. aeruginosa during static coculture relative to monoculture but does impact spatial organization and susceptibility to tobramycin. These data may be particularly meaningful for coculture studies, including those in animals, as bacterial numbers (fitness) are often the primary data used to identify interactions and assess the relevance of specific pathways on bacterial interactions. We propose that assessing spatial organization of communities may be as informative as assessing fitness and that the use of straightforward pipelines for quantifying spatial structure will be critical for understanding the functions of human-associated microbial communities.

While our study focused on laboratory strains of P. aeruginosa and S. aureus, it is clear that the genotype of the strains used can impact relative fitness during coculture (20, 40, 57). Although we anticipate that relative fitness might be impacted by the use of other strains, as P. aeruginosa PA14 is highly lytic for S. aureus LAC under well-mixed conditions, their survival in static SCFM2 indicates that this model will likely promote coexistence for multiple genotypes, even those that are highly antagonistic. Finally, it was previously suggested that SCFM2 does not support robust coexistence of P. aeruginosa and S. aureus, even under static growth conditions (41). However, in the previous study, the static assay quantified bacteria that remained attached to the well of a 96-well plastic dish after vigorous washing, ultimately demonstrating that P. aeruginosa was ∼100-fold more prevalent than S. aureus after 12 h. The likely reason these results differ from ours is that most of the bacteria in SCFM2 grow as suspended aggregates and planktonic cells (17, 44), and we have not focused on the bacteria attached to surfaces, as our goal is to model human infection. In addition, the fact that the previous study observed ∼10^7^ bacteria attached to the plastic surface at 12 h (41) reveals that only about 1% of the total number of bacteria in the coculture were growing attached to plastic, as the carrying capacity of P. aeruginosa in SCFM2 is >10^9^ CFU/ml (Fig. 1A and B). Ultimately, our results provide strong evidence that static growth in SCFM2 supports coexistence of P. aeruginosa and S. aureus and provides the opportunity to study interactions between these common coinhabitants of human infections.

## MATERIALS AND METHODS

### Strains, media, and growth conditions.

Prior to use, strains were streaked on tryptic soy agar plates (Sigma), inoculated into tryptic soy broth, and grown overnight at 37°C with shaking at 250 rpm. Wild-type P. aeruginosa strain PA14 and PA14 Δ*pqsL* were fluorescently labeled with the GFP-expressing plasmid pMRP9-1 (58) and maintained with 100 μg/ml of carbenicillin. S. aureus LAC was fluorescently labeled by moving pHC48 (59), containing DsRed under the control of the *sarA* promoter, from RN4220 by phage transduction. S. aureus carrying pHC48 was cultured with 10 μg/ml of chloramphenicol to maintain the plasmid.

### Construction of the P. aeruginosa PA14 *pqsL* deletion mutant.

A *pqsL* deletion mutant was constructed in PA14 by removing the gene *pqsL* by homologous recombination. The knockout construct was made by amplifying approximately 1-kb DNA fragments upstream and downstream of *pqsL* with overlapping sequences and ligating these fragments into pEXG2 (Promega) using Gibson assembly to form pEXG2pq. Primer sequences to PCR amplify the *pqsL* upstream region were tcggtacccggggatcctctGGTGTTCAACGTGGTCCC and aggaacgctcGCAGCCGTTGATCAGTAC and for amplification of the *pqsL* downstream region were caacggctgcGAGCGTTCCTATCAGCCG and catgcctgcaggtcgactctGTGTTCCTCAATCTGCTGC (capital letters indicate bases that anneal to the P. aeruginosa target region, and lowercase corresponds to overhang sequences used for Gibson assembly). For linearizing pEXG2, we used the primers GCTTTACATTTATGCTTCC and ATGATCGTGCTCCTGTCG. The fragments were combined using Gibson assembly, and the resulting ∼7-kb plasmid (pEXG2pq) was purified and transformed into Escherichia coli DH5α using the transformation and storage solution (TSS) method (60) and selected on 15 μg/ml gentamicin. The plasmid was then purified and transformed into E. coli SM10 λ*pir* using the TSS method (60) and selected on 15 μg/ml gentamicin. The knockout vector (pEXG2pq) was conjugated into P. aeruginosa as previously described (61). E. coli was counterselected using *Pseudomonas* isolation agar plates (Sigma), and P. aeruginosa recombinants were selected with 60 μg/ml gentamicin. Forty-eight colonies were screened for sensitivity to sucrose. Allelic replacement was confirmed by PCR and phenotypically by identifying colonies that exhibit autolysis (62).

### Complementation of P. aeruginosa PA14 Δ*pqsL*.

*pqsL* was amplified with the Expand long-template PCR system (Sigma) from P. aeruginosa PA14 chromosomal DNA using the forward primer 5′-GAATTCGGAACGACACGGAGACTCATCC-3′ and reverse primer 5′-GAGCTCAGCCGCGCGGAGC-3′. The 1,238-bp amplicon was ligated into the TOPO cloning vector using the TOPO TA cloning kit (Thermo Fisher) to create pTOPO-pqsL. *pqsL* was then removed from pTOPO-pqsL by EcoRI digestion and cloned into EcoRI-digested pBBR1MSC-5 (63). In the resulting plasmid (pBBR1-pqsL), *pqsL* is oriented such that it is transcribed from the *lac* promoter.

### Growth in SCFM2.

Overnight cultures of P. aeruginosa and S. aureus were subcultured in SCFM (21) until they reached exponential phase (optical density at 600 nm [OD_600_] of 0.3 to 0.6). Cultures were then washed and concentrated in prewarmed SCFM without antibiotics to an OD_600_ of 1.0. These cultures were then used to inoculate each bacterium into SCFM2 at an OD_600_ of 0.05 in mono- or coculture. A 300-μl portion of inoculated SCFM2 was then placed into wells of an 8-well optical chamber (Nunc Lab-Tek chambered cover glass) and incubated statically or well mixed at 37°C. Well-mixed cultures included a magnetic stir bar (1.5 by 8 mm) in each of the wells rotating at 250 rpm. Growth was assessed at 4 h intervals (4, 8, 12, and 16 h) using dilution plating with P. aeruginosa- and S. aureus*-*selective media, *Pseudomonas* isolation agar and Baird-Parker agar, respectively.

### Disc diffusion assays.

For the supernatant assays, P. aeruginosa cultures were grown as described above in SCFM2. After 16 h, cultures were centrifuged at 5,000 rpm for 10 min, and supernatants were filtered through a 0.45-μm syringe filter and placed on ice. Overnight cultures of S. aureus were spread on brain heart infusion (BHI) plates using sterile swabs, 6-mm filter paper discs were placed onto the agar, and 10 μl of either P. aeruginosa supernatant or SCFM2 (as a control) was added to each disc and allowed to dry at room temperature. The zone of inhibition was measured after 24 h of incubation at 37°C. The reported normalized zone of inhibition was calculated by measuring the area of inhibition created by each culture condition and divided by the respective growth yield at that culture condition. To assess lysis by P. aeruginosa cells, instead of supernatant being added to a disc, 5 μl of planktonic BHI-grown P. aeruginosa (OD_600_ = 0.5) was added.

### Tobramycin susceptibility assay.

S. aureus was inoculated as described above in SCFM2 in mono- or coculture with P. aeruginosa, grown statically for 3 h, then treated with 256 μg/ml tobramycin for 2 h. Surviving bacteria were quantified by dilution plating with P. aeruginosa- and S. aureus*-*selective media, *Pseudomonas* isolation agar and Baird-Parker agar, respectively.

### CLSM imaging.

The SCFM2 culture method described above was used for imaging. Three wells (S. aureus monoculture, P. aeruginosa monoculture, and coculture) per optical chamber were used for each replicate imaging experiment. Each experiment imaged a single position in each well, once per hour. All images were acquired with a Zeiss LSM 880 CLSM utilizing Zen image capture software. Detection of DsRed-expressing S. aureus cells was performed with an excitation wavelength centered at 587 nm and an emission wavelength centered at 610 nm. Detection of GFP-expressing cells was performed using an excitation wavelength centered at 488 nm and an emission wavelength centered at 509 nm. All images were acquired using a 63× oil-immersion objective. All data were stored as 1,024- by 1,024-pixel slices in stacks of 91 8-bit images. Each voxel is 0.264 by 0.264 by 0.44 μm^3^.

### Image thresholding.

Confocal images were exported as a tiff stack and thresholded using MATLAB (Simulink). A threshold was identified for each image stack using Otsu’s method (64). For each channel, the final threshold for all images was identified by calculating a trend line over time across all calculated thresholds and using the value at the median time point.

### Calculating aggregate size and histograms.

Binarized image stacks were imported as a 3D matrix and segmented using the bwconncomp function (MATLAB R2019a; Simulink), finding connected voxels with 18-level connectivity (identifying voxels that touch at one of their faces or edges). The size of each object was mapped from voxels to cubic micrometers, and a histogram with a 5-μm^3^ bin size was created using a custom script in R (version 3.6.1).

### Determination of single versus multispecies aggregates.

To identify multispecies aggregates, cocultured binarized image slices with S. aureus in the red channel and P. aeruginosa in the green channel were converted to grayscale images using im2bw function and segmented using the labels function to identify connected pixels in 2 dimensions of bacterial biomass, independent of species (MATLAB R2019a; Simulink). Those bacterial segments create a bacterial objects matrix. Two more matrices were created; one for P. aeruginosa (green channel) and another for S. aureus (red channel). The bacterial matrix was then combined with either the green or red matrix from the same image to characterize the composition of bacterial segments in regard to species-specific segments. Bacterial aggregates composed of more than one channel were considered multispecies aggregates.

### Calculating proportional occupancy and enrichment distance.

To determine proportional occupancy, binarized image stacks were analyzed using a custom pipeline developed in R (publicly available at https://jupabago.github.io/PaSaProject/). Briefly, a focal voxel in the 3D image was picked at random and the voxels of a specific channel that were located within a spherical distance interval away (between radius 1 and radius 2) from the focal voxel were counted. Proportional occupancy was calculated by multiplying the number of voxels within a distance interval by the size of each voxel and dividing by the total volume of the spherical shell bound by that distance interval: (number of voxels in distance interval × voxel volume)/total volume of interval.

Proportional occupancy was obtained for 5,000 random focal voxels per image, starting from a distance of 1 μm away from each focal voxel and continuing for 30 μm using 1-μm distance intervals. When the focal voxel picked at random was located closer than 30 μm to the edge of the image, the proportional occupancy was corrected by using the volume of shells from a capped sphere instead of spherical shells. Proportional occupancy was calculated using S. aureus as a focal point and using S. aureus (control) or P. aeruginosa as surrounding cells. For the 5,000 random focal voxels, a histogram of proportional occupancy values was produced at each distance. The proportional occupancy was calculated for each distance interval as the weighted median, and the distance interval with the highest weighted median is the enrichment distance.
