# Supplementary figures and images for: A Pseudomonas aeruginosa Antimicrobial Affects the Biogeography but Not Fitness of Staphylococcus aureus during Coculture
Source: mBio. 2021 Mar 30;12(2):e00047-21. doi: 10.1128/mBio.00047-21 (PMC8092195; doi:10.1128/mBio.00047-21)

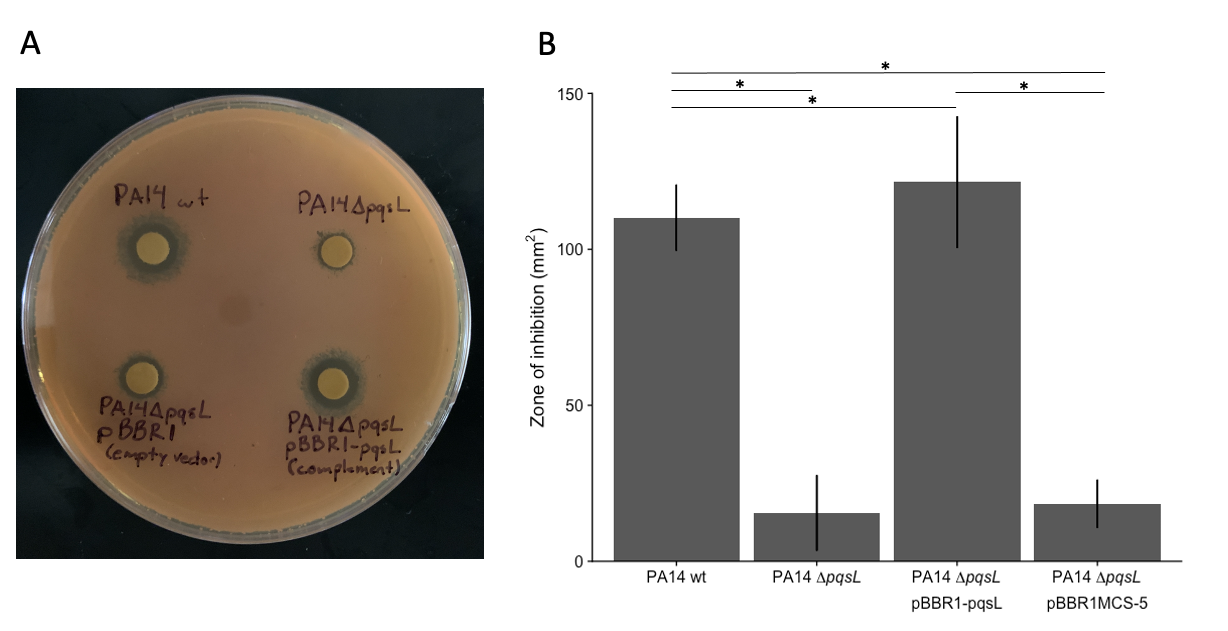

Supplement: FIG S1 [file mBio.00047-21-sf001.tif]
